# Challenges in Obstructive Sleep Apnea Management in Elderly Patients

**DOI:** 10.3390/jcm13247718

**Published:** 2024-12-18

**Authors:** Aude Joskin, Marie Bruyneel

**Affiliations:** 1 Department of Pulmonary Medicine, Saint-Pierre University Hospital, Brussels, Belgium and Université Libre de Bruxelles, 1000 Brussels, Belgium; aude.joskin@stpierre-bru.be; 2 Department of Pulmonary Medicine, Brugmann University Hospital, Brussels, Belgium and Université Libre de Bruxelles, 1020 Brussels, Belgium

**Keywords:** obstructive sleep apnea, elderly, continuous positive airway pressure, cognition, OSA phenotypic traits, personalized sleep medicine, patient-centered sleep diagnostics

## Abstract

With the aging of the population, obstructive sleep apnea (OSA) in elderly patients is now more commonly seen in clinical practice. In older people, sleepiness is less marked than in younger patients, but insomnia symptoms are more common. Comorbidities are numerous and related to cardiometabolic and cognitive conditions. Polygraphy can be used to establish the diagnosis in the vast majority of cases, but polysomnography is indicated in cases of comorbid sleep disorders. Continuous positive airway pressure (CPAP) remains the cornerstone of treatment, but compliance decreases with age, especially in those over 80, and when cognitive disorders are also present. In these patients, CPAP can be beneficial in terms of nighttime symptoms, sleepiness, mood, and cognition but can also prevent cardiovascular and cerebrovascular disorders, especially in severeOSA patients. For this reason, we should offer this treatment to elderly patients and devise strategies to support them with treatment difficulties (e.g., therapeutic education, adapted masks, and telemonitoring). In the future, we need prospective studies to help identify elderly patients who will gain the greatest long-term benefit from treatment. Dedicated sleep testing, OSA severity markers, and specific questionnaires need to be developed in this older, but large, OSA population.

## 1. Introduction

With the aging of the population, obstructive sleep apnea (OSA) in elderly patients (chronological age of 65 years old or more) has become more frequently seen in clinical practice. Aging is one of the most important risk factors for OSA. In Europe, elderly people represented 20.3% of the population in 2019, and this proportion is expected to grow to 30% by 2070. Life expectancy has increased from 76.9 years in 2000 to 81.5 years today, and very old people (>80 years) represent 6.1% [1].

The increasing size of the elderly population suggests that the prevalence of OSA is also likely to rise. A recent Swiss epidemiologic study reported that 60% of men and 35% of women older than 60 years of age exhibit at least moderate OSA on sleep testing (apnea–hypopnea index (AHI) > 15/h of sleep) [2]. The treatment of OSA in elderly patients can contribute to well-being and healthy aging.

Mechanisms that may explain the observed associations between aging and OSA include increased upper airway (UA) resistance during sleep, decreased pharyngeal muscle activity, lateral and antero-posterior UA dimension, altered UA muscle reflexes, and the occurrence of central respiratory events in older adults [3]. Indeed, the usual OSA risk factors, such as obesity, are less predominant in elderly patients [4].

OSA contributes to the occurrence of cardiovascular disease (CVD), type 2 diabetes, and cognitive disorders, even in the elderly, but the physiologic effects of aging also have significant interplay [5,6,7]. Comorbidities are frequent in elderly patients, including hypertension, chronic obstructive pulmonary disease (COPD), heart failure, cancer, and dementia [8]. However, older OSA patients appear to suffer fewer health consequences from OSA than younger patients [3]. This remains controversial, however, as it has also been shown, in a large American retrospective study on Medicare beneficiaries, that the risk of hospitalization in older patients (>65 years) suffering from CVD was doubled in the year prior to OSA diagnosis compared to non-OSA patients, suggesting that the impact of OSA is still significant in this population [9].

In addition, a longitudinal population-based study (HypnoLaus) reported that untreated OSA and nocturnal hypoxemia were associated with a 5-year cognitive decline in 358 patients > 65 years of age [10]. The relationship between OSA and neurodegenerative disorders is probably bidirectional. On the one hand, intermittent hypoxia and disturbed sleep quality seem to favor Alzheimer’s disease, but the impact on cognition and mild cognitive impairment remains controversial [10,11]. On the other hand, central dysfunction of the respiratory structures of the brainstem caused by neurodegenerative disorders such as Parkinson’s disease can induce sleep apnea [12].

The difficulty of defining the impact of OSA on health consequences may potentially be related to the use of AHI as the central metric for assessing severity. Indeed, AHI is not well correlated with symptoms or with comorbidities [13]. For example, AHI does not consider the amount of hypoxemia provoked by obstructive respiratory events, the length of the events, and other pathophysiological consequences. Indices that assess (intermittent) hypoxia, such as hypoxic burden, are better correlated with CVD consequences [14] and provide a promising alternative tool for the assessment of OSA severity in elderly patients. As older people may be less susceptible to brain oxidative stress [5], the use of specific biomarkers (e.g., microRNAs related to hypoxia, IL-6, IL-8, high-sensitivity CRP, TNF-α, homocysteine, and cysteine) should also open up future possibilities for the impact assessment of OSA in elderly patients [15].

The last decade of research has been centered around identifying OSA phenotypes/endotypes to support a personalized medicine approach in OSA. Most studies on phenotypes and cluster analyses have focused on middle-aged (mean age 52–59), mainly male (70–95%) patients [16,17,18,19,20,21]. The treatment of OSA is focused on two potential benefits: the regression of OSA-related symptoms (e.g., sleepiness, insomnia, and decreased cognitive function) and improvements in patients’ prognoses with regard to cardiovascular comorbidities (e.g., arterial hypertension, atrial fibrillation, and stroke). When focusing on elderly patients, the maintenance of adequate cognitive function should also be considered.

As we face the increasing number of patients >65 years of age who are attending sleep laboratories (12% in our center), there are numerous challenges to be addressed in the European elderly population: the identification of OSA-related symptoms, accurate diagnoses, the impact of OSA treatment on symptoms, comorbidities, and mortality. In this review, we will discuss these challenges and review the current evidence for best management.

## 2. Clinical Features and Diagnostic Methods for Assessment of OSA in Elderly Patients

The clinical characteristics of OSA in elderly patients are different from those observed in younger patients. First of all, in healthy older adults, normal physiologic sleep changes induce increased wake after sleep onset, lower total sleep time and sleep efficiency, and decreased slow-wave sleep. Insomnia complaints are frequent in older and very old people [22]. In contrast, snoring decreases with aging [23]. Sleep disruption, fatigue, nocturia, napping, and cognitive dysfunction are common clinical features that appear with aging [24]. This makes it difficult to assign complaints regarding sleep quality to potential OSA syndrome (OSAS) when they may simply be due to the effects of aging. If daytime symptoms are present, this can lead to suspicions of OSAS, but fatigue and sleepiness can also be related to the higher levels of comorbidities and medications (particularly psychotropes) that are frequently observed in elderly patients. OSA-related excessive daytime sleepiness (EDS) is less marked in older patients than in younger patients, but insomnia symptoms are more common [3,25].

Another confounding factor is the potential presence of central sleep apnea (CSA) in elderly patients. In this population, congestive heart failure is common, reaching up to 8.3% [26]. It is estimated that 34–69% may have CSA and Cheynes Stokes respiration (CSR). The symptoms associated with this condition (insomnia, fatigue, frequent awakenings, and poor sleep quality) may overlap with those of OSAS [27]. This underlines the need to properly document sleep-disordered breathing in this population.

Gender differences can also lead to different clinical pictures in the elderly [28]. In women, symptoms differ from those observed in men: while men report sleepiness and apnea, nocturnal choking, morning headaches, fatigue, insomnia symptoms, and nocturia are more frequent in women [29]. The prevalence of OSA in females doubles after menopause and, in contrast to men, is independent of obesity. Peak prevalence is observed at age 65 years, 10 years later than in males [29]. A lower loop gain and higher apnea threshold seem to protect premenopausal females from OSA [30]. Women diagnosed with OSA are, therefore, older at the time of initial diagnosis, but they are also diagnosed with less severe OSA than their male counterparts: they have lower AHIs during REM and non-REM sleep and a lower AHI in the supine position. The result is milder sleep apnea diagnoses in women [31].

There are no specific or validated tools intended to assess the symptoms of OSA in elderly patients (e.g., the Epworth Sleepiness Scale (ESS)) [32]. As a consequence, OSAS is still underdiagnosed in older people [9,33]. Braley et al., in a sample of community-dwelling Medicare beneficiaries (>65 years old), highlighted via screening questionnaires that 56% were classified as high-OSA-risk patients, but only 8% were previously investigated. Among these patients, 94% were finally diagnosed with OSA [33]. In a large American retrospective study on Medicare beneficiaries, Kirk et al. reported the prevalence of undiagnosed OSA in patients older than 65 years of age suffering from CVD. This prevalence reached 13.5% and was associated with a two-fold increase in hospital admissions compared to patients with cardiovascular disease without OSA [9]. Typical comorbidities (e.g., atrial fibrillation and heart failure), rather than symptoms, should be the trigger for sleep investigation in a significant proportion of elderly patients. In patients with other frequent comorbidities (e.g., CVD, glaucoma, and neurocognitive disorders), case finding can be proposed according to symptoms. Indeed, the usual OSAS presentation (i.e., obesity, naps, loud snoring, and severe OSA ± marked hypoxemia) is still a valid reason for suspecting the disorder in older patients and has been well documented in cluster analyses of OSA phenotypes [15]. Screening for OSA in adults is not currently recommended [34].

A major challenge in elderly patients is choosing the right diagnostic procedure to detect respiratory events and to be able to quantify them correctly. OSAS diagnosis is based on criteria that combine symptoms, comorbidities, and polysomnography (PSG) recordings that indicate the presence of obstructive respiratory events. In-laboratory PSG continues to be the reference diagnostic method [35], but polygraphy (PG), generally performed at home, has become widely accepted and can be used for OSA diagnosis in patients with a high pre-test probability of moderate-to-severe OSA [36]. Practically, despite some limitations (e.g., a lack of electroencephalogram means that a correct assessment of total sleep time and arousals is not possible), PG is used worldwide for OSA diagnosis as it increases the accessibility of sleep testing and hastens treatment initiation. However, in elderly patients, an increased prevalence of stroke and heart failure is observed, resulting in more frequent CSA and CSR [37]. Even if CSA and CSR can be easily recognized on PG, full PSG remains the gold standard not only for the diagnosis of CSA-CSR but also to differentiate it from other sleep disorders. This is indeed clinically relevant, as insomnia or movement disorders are associated with arousals that induce CSA in sleep–wake transitions [27,36]. Sleep disruption, heart failure, stroke sequelae, COPD-OSA overlap, REM behavior disorder, polymedication, and other neuro/sleep comorbidities should encourage the use of PSG rather than PG as the first option in some elderly patients [38].

## 3. After Diagnosis

Once the diagnosis has been made, the question is, if the investigation was triggered by symptoms, are those symptoms related to OSAS? When other causes of similar symptoms are reasonably excluded based on anamnesis, medical history, and a review of medications, the next step is to try OSAS treatment and to observe symptom changes.

According to Swedish consensus, OSA specialists recommend starting OSA treatment (moderate, strong, or very strong indication) in patients > 65 years only if patients are symptomatic or suffer from comorbidities of interest (e.g., hypertension atrial fibrillation, ischemic heart disease, heart failure, stroke, diabetes, and obesity) that are not well controlled; or if they are asymptomatic, having an AHI of ≥30; or if they are symptomatic, having an AHI of ≥5 [39].

CPAP is the main reference treatment for moderate-to-severe OSAS (AHI > 15/h of sleep). However, only 40–85% of patients are compliant with CPAP. Factors associated with poor compliance are age, poor socioeconomic status, an active smoking status, a low severity of OSA, and fewer symptoms (e.g., daytime sleepiness). Psychosocial factors (e.g., marital status/bed partner involvement) and side effects also play a significant role in compliance [40]. Local organization of care can also have a significant impact on compliance. For example, in Belgium, OSA care is organized into hospital sleep laboratories recognized by national health insurance. The system imposes multidisciplinary management, regular follow-up by sleep specialists, and proof of CPAP compliance for continuation of treatment. In this setting, CPAP termination rates are low: 8% yearly [41]. In France, where CPAP treatment follow-up is mainly managed by general practitioners and home care providers, nearly one-quarter of patients stop therapy in the first year after CPAP initiation [42]. Telemonitoring, associated with a closer follow-up of patients, especially at treatment initiation, has been shown to improve compliance [43].

A diagnostic therapeutic trial (with CPAP) is becoming more and more commonly used to link sleep symptoms to OSA disorder (this is also true in younger patients), as the usual metrics for OSA (particularly AHI) have a limited value for capturing the full clinical picture of OSA [44]. If the OSAS diagnosis is suspected to be due to comorbidities, the impact on the few potential symptoms can be highly variable, and CPAP should be attempted to improve comorbidities [45]. There are some alternatives to CPAP therapy for older patients suffering from OSAS but fewer than those for younger patients.

Mandibular advancement devices (MADs), indicated for mild-to-moderate OSA (AHI > 5/h of sleep), are also challenging, with 63% long-term compliance [46]. Side effects are often experienced by patients, such as occlusal changes, hypersalivation, temporomandibular joint pain, and discomfort. Factors associated with MAD compliance are effectiveness (AHI reduction > 50%) and complete symptom resolution (e.g., snoring, EDS, nocturia, and headache). Patients that exhibit an “arouser” pattern on PSG, which means that arousals during sleep are triggered by respiratory events, are also good responders [46,47]. MADs are often contraindicated due to poor oral health and edentulism in elderly patients but can be proposed in some cases. In this age group, patients using MAD are generally long-term users who started MAD treatment years before. In a large French ORCADES study, in which patients were reassessed five years after the start of MAD use, the oldest patient was 61 [48]. In well-selected cases, hypoglossal nerve stimulation (HNS) may also be an option [20]. Positional therapy provides an additional alternative, but positional OSA generally affects younger patients and rarely affects women [29,49].

Weight loss through bariatric surgery can be offered to obese patients. However, higher complication and mortality rates are observed after surgery in patients > 70 years of age [50]. New effective drugs have recently been developed for the treatment of obese patients. GLP-1 receptor agonists and gliflozins have been shown to induce weight loss and improve cardiometabolic dysregulation in patients with metabolic syndrome and type 2 diabetes. Indeed, liraglutide seems to achieve direct anti-atherosclerotic action by decreasing plaque formation and progression [51,52]. Tirzepatide, a dual GIP/GLP-1 receptor co-agonist, has been shown impressive efficacy on OSA in a recently published randomized controlled trial (RCT). Among moderate-to-severe obese OSA patients, tirzepatide reduced AHI by 51–59%, body weight by 18–20%, and also reduced systolic blood pressure after 52 weeks of treatment. This drug can also improve renal function and hepatic steatosis and is, therefore, potentially very promising in the elderly and comorbid OSA population [53].

Other pharmacological treatments can also be used to treat residual EDS in CPAP users (6–15% of patients) or in sleepy OSA patients who refused or did not tolerate CPAP. Pitolisant is a selective histamine H3 receptor antagonist with strong wake-promoting effects and has been shown to reduce EDS in both patient categories with a very good safety profile. Solriamfetol, a dual dopamine/norepinephrine reuptake inhibitor, has been studied for persistent EDS in CPAP-treated OSA patients, with positive impacts on ESS and a good safety profile. Finally, modafinil and armodafinil, both dopamine reuptake inhibitors, are also effective for residual EDS in CPAP users and in sleepy untreated OSA patients, but these medications tend to increase systolic and diastolic blood pressure, and as a result, their indication is limited [54].

## 4. CPAP Treatment in Elderly Patients

A limited number of studies related to CPAP treatment have focused on elderly patients. Most of these studies have been conducted by Martinez-Garcia et al., in collaboration with the Spanish Sleep network. The largest cross-sectional study, conducted in Spain, reported that 70% of OSAS patients > 65 years of age were treated with CPAP [55]. Only three large multicentric RCTs have been performed in patients aged > 65 or >70 [56,57,58]. Outcomes were assessed at 3 months in two of the trials and at 12 months in the other trial. The included patients had a diagnosis of moderate-to-severe OSAS (*n*= 278, mean oxygen desaturation index: 28.7/h), severe OSAS (n = 224, mean AHI: 50/h), or moderate OSAS (*n* = 145, mean AHI: 22/h). The results from these studies are discussed below.

However, it should be noted that in the oldest patients, over 80 years of age, compliance of >4 h/night is so low (23.8%) that it is difficult to assess any effect of CPAP treatment in this subgroup [59].

### 4.1. Impact of CPAP Treatment on Symptoms in Elderly Patients

#### 4.1.1. Excessive Daytime Sleepiness

Improvements in daytime sleepiness were observed in all three trials. CPAP treatment was associated with a significant decrease in ESS score (to a normal score) in all patients [57]. This was confirmed in an RCT that focused on moderate CPAP-treated OSA [55] and was also maintained at 12 months [56]. In this study, objective sleepiness was measured by the OSLER test and significantly decreased at 3 months but not at 12 months.

This improvement was also confirmed in a large prospective pan-European ESADA cohort [25], in which 687 CPAP-treated patients, aged 70–79, showed a decrease in ESS from 9.6 to 7.2 even when no pathologic EDS was reported at the baseline.

#### 4.1.2. Quality of Sleep

In a study by Martinez-Garcia et al., nighttime and daytime symptoms, assessed by the Quebec Sleep Questionnaire (QSQ), improved significantly with CPAP [55]. In a study from Ponce et al., which included less-severe patients (moderate CPAP-treated OSA), only nocturnal symptoms and emotions were improved in the QSQ [58].

In addition, in a recent observational prospective study, the objective total sleep time was also improved with CPAP, as was nocturia [60].

#### 4.1.3. Anxiety and Depression

In RCTs in elderly patients, about 20% of elderly OSAS patients suffered from anxiety and/or depression at the time of diagnosis. These symptoms improved in a subgroup of 115 severe CPAP-treated OSAS patients [57], but this outcome was not observed in moderate-OSAS patients [58].

#### 4.1.4. Cognitive Dysfunction

CPAP resulted in a significant improvement in digit symbol score related to working memory in the aforementioned subgroup of 115 severe-OSAS CPAP-treated patients [57]. However, this outcome was not observed in elderly patients with moderate OSA [58], and no cognitive benefit of CPAP treatment was reported in a study from McMillan et al., despite assessment by four different tests [56].

In prospective studies, a benefit of CPAP was observed in 98 OSAS patients on mild cognitive impairment occurrence (delayed by 10 years) [61], and a study by Liguori et al. showed normalization of CSF beta amyloid 42 and t-tau/beta amyloid 42 after one year of CPAP in OSA patients (mean age: 72), leading clinicians to consider whether OSA might be a reversible risk factor for dementia [62].

### 4.2. Impact of CPAP Treatment on Comorbidities and Mortality

According to data from RCTs on CPAP treatment in elderly OSA patients, effects on blood pressure are somewhat mixed. No differences were observed in systolic and diastolic blood pressure measurements when comparing elderly CPAP-treated and control groups at 3 months in a study from Martinez-Garcia et al. [57]. However, McMillan et al. reported that systolic blood pressure was improved (−3.7 mm Hg) at 12 months [56]. Of note, the reported effects of CPAP on systolic and diastolic blood pressure were relatively low (−2.5 and −2 mm Hg, respectively) in a meta-analysis performed on data from younger patients [45].

To date, RCTs have failed to show a positive impact of CPAP treatment on mortality in large series of adult OSA patients (SAVE study, ISAAC study, and RICADDSA study) [63,64,65]. However, post hoc analysis showed that patients with high hypoxic burden and adequate compliance benefit from CPAP for secondary cardiovascular prevention.

The effect of CPAP in elderly patients has been evaluated in observational and retrospective studies [66]. In a prospective observational study from Martinez-Garcia et al. in patients > 65 who were followed for 69 months, severe-OSAS patients who refused CPAP or were non-compliant had higher excess mortality related to stroke, heart failure, and coronary artery disease compared to those with mild-to-moderate untreated OSAS (HR = 2.25) [67].

Another study showed that, in 155 severe-OSA patients over 80, the mean survival was 91 months in CPAP-treated patients vs. 52 months in untreated patients. Mortality was mainly related to cardiovascular and infectious disorders, regardless of the use of CPAP [68].

In moderate OSA, the effect of CPAP on cardiovascular (CV) events or mortality has not been demonstrated [67,69]. This observation probably reflects the fact that the cut-off AHI used to establish the diagnosis of significant OSA must be raised in elderly patients.

Secondary prevention of stroke represents a specific purpose of CPAP treatment. In patients with a history of stroke, with at least moderate OSAS, CPAP use significantly decreased the risk of recurrence or CV mortality in a prospective study of 101 patients aged 68.5 years on average [68] who were followed 2 years post-stroke. The same result was observed by Martinez-Garcia et al. in 166 patients who were followed for 7 years after a stroke episode [67]. The use of CPAP in moderate-to-severe OSA (mean AHI = 41/h) versus no CPAP (mean AHI = 35/h) significantly reduced the future risk of a CV event and stroke to the risk of non-OSAS patients. The mean age of the patients was 71 years in CPAP users and 76 years in non-users.

Discrepancies among the observed effects of CPAP on CVD and mortality have been previously described. In older patients, one explanation could be the fact that, above age 65, when a CV event occurs, the damage is already done, and the deleterious OSA effect may not be relevant. Moreover, the vast majority of this elderly population suffers from other additional CV risk factors (e.g., metabolic syndrome and sedentarity). The hypothesis of ischemic preconditioning has been also widely debated. Through the activation of adaptive mechanisms involving coronary artery occlusion and reperfusion to overcome nocturnal intermittent hypoxia secondary to OSA, it can be hypothesized that moderate OSA could prevent subsequent major CV events instead of producing them. An initial study supporting this hypothesis showed that untreated sleep apnea conferred a survival benefit in older patients [70].

### 4.3. Challenges Associated with CPAP Use

#### 4.3.1. Barriers to CPAP Treatment

Aging is associated with frailty, chronic pain, cognitive decline, disrupted sleep, and nocturia [24]. Many older people spend more than 10 h/day in sedentary behavior. This is associated with lower cognitive function [71] and lower upper body muscle strength [72]. Impaired prehension dexterity can be a real barrier for CPAP use as the inability to place the mask correctly will result in non-effective treatment, major leaks, and excessive noise, compromising CPAP therapy. Diuretic treatment can increase nocturia and make the treatment more difficult. Increased risk of falls related to the presence of the CPAP device/tube can also contribute to difficulties.

#### 4.3.2. Compliance with CPAP

Obtaining adequate compliance remains a major goal when starting CPAP. The first challenge is to obtain acceptance. Data reported in a series of 115 patients suffering from severe OSAS and aged > 65 years revealed that acceptance is satisfactory—up to 70% [73]—but is lower than in younger patients [57]. However, even among older acceptors, 37% of patients stop treatment within the first year, much more often than in younger patients [41].

Regarding compliance, the data are conflicting. Martinez-Garcia et al. observed that compliance was <4 h in 30% of 115 elderly CPAP-treated patients [59]. Martinez-Garcia et al. also showed that compliance falls with increasing age, with less than 50% of patients using CPAP for at least 4 h/night after 60 months in the over-80 subgroup [59]. The mean use drops to 2.9 h/night in this age category.

In an RCT from McMillan et al., which included moderate-to-severe-OSAS patients, among 140 patients randomly assigned to CPAP, 120 (86%) and 99 (71%) self-reported they were still using CPAP at 3 months and 12 months, respectively. Usage data for CPAP were obtained for 117 patients at 3 months, showing a very low median usage duration of 1 h 52 min per night, and for 102 patients at 12 months, showing a median usage duration of 2 h 22 min per night [56].

However, in the ESADA cohort [40], CPAP follow-up data on the 687 patients of the 70–79 age group showed that their compliance was similar to that of younger patients, estimated at 6h/night. Poorer CPAP adherence was associated with higher score on the Clinical Global Impression Severity Scale, reflecting lower overall function. The same was observed in a multicenter study by Lopez-Padilla et al. in which adequate compliance was reported in 60% of patients [69].

A recent French study (S.AGES) explored CPAP adherence in 262 very old patients (aged 77–88 years) at 5 months. Eighty-six percent were adherent to CPAP. Conversely, adherence was decreased in elderly patients with dementia and poor mental health [60].

## 5. Conclusions

The current general belief is that elderly patients probably derive poor benefit from CPAP therapy. In the view of many physicians, the difficulties related to CPAP use in elderly patients likely outweigh the benefits, and in this regard, it is easier to leave the patient untreated than to propose a treatment that will need a lot of support and close monitoring and will be time consuming for sleep laboratory technical teams.

In this review, we have shown that CPAP treatment can be beneficial in older OSA patients in terms of nighttime symptoms, sleepiness, mood, and cognition and also in prevent cardiovascular and cerebrovascular disorders, especially in severe-OSA patients. This means we must offer this treatment to elderly patients.

The 2016 recommendations from the International Geriatric Sleep Medicine Task Force, advises the following for elderly patients [74]:

- CPAP should be used routinely for the treatment of sleep breathing disorders in older persons and in the frail elderly, particularly those with stroke but without major heart failure with an ejection fraction of ≤45%.
- While additional RCTs are needed in patients with Alzheimer’s or Parkinson’s disease, as well as other frail elderly patients, these patients do tolerate CPAP, and treatment should be considered.

From this review of the current literature, and according to these guidelines, we can extract guidance to select treatment in elderly patients.

It is currently clear that CPAP can be beneficial in patients > 65 years of age who are suffering from severe OSA, considering that this patient category is often comorbid but not frail.

On the other hand, the prescription of CPAP in elderly OSA patients aged > 75 years (and more particularly ≥ 80 years) should be individualized, targeting sleepy patients and patients with a history of stroke, as treatment-related difficulties (e.g., cognitive dysfunction) might outweigh the benefits and as short-term CPAP compliance is particularly low in this population. In the case of CPAP intolerance, alternative treatments can be attempted. Tirzepatide seems particularly promising, as it achieves weight loss and OSA severity reduction and may be active on cardiovascular, renal, and liver comorbidities. It also seems acceptable to leave a very old patient untreated.

In the future, as is the case with younger patients, we need prospective studies that help to identify older patients who will achieve the greatest long-term benefit from CPAP treatment. Dedicated sleep testing, the identification of OSA severity markers, and specific questionnaires need to be developed in this particular—but large—OSA population. Cost effectiveness studies would provide additional data to place CPAP treatment in a population-based context. Finally, as CPAP remains difficult to use, particularly in very old patients, alternatives to CPAP need to be developed.
